# Simultaneous musculocutaneous nerve entrapment and radial nerve traction injury after open subpectoral biceps tenodesis: a unique case demonstrating a rope-effect mechanism

**DOI:** 10.1016/j.xrrt.2026.100806

**Published:** 2026-06-17

**Authors:** Vicente Pellicer-Garcia, Maria del Carmen Castro Copete

**Affiliations:** a Department of Orthopedic and Trauma Surgery, General University Hospital Virgin of the Lilies, Alcoy, Spain; b Department of Radiodiagnosis, General University Hospital Virgin of the Lilies, Alcoy, Spain

## Introduction

Open subpectoral biceps tenodesis is a well-established treatment for long head of the biceps^1^ (LHBs) pathology, providing reliable outcomes and low complication rates.^2^, ^3^, ^4^, ^5^, ^6^ However, the musculocutaneous nerve (MCN) lies close to the subpectoral fixation site. Cadaveric studies show that arm positioning—particularly external rotation—significantly alters MCN proximity to the humerus and drill trajectory.^2^ Most reported nerve complications are transient neuropraxias related to traction or retractor pressure.^6^, ^7^, ^8^, ^9^

Only 1 published case has identified true structural MCN entrapment beneath a tenodesed tendon.^4^ Radial nerve injury after biceps tenodesis is exceedingly rare and usually results from traction or drill misplacement.^1^ To date, no case has described simultaneous MCN entrapment with radial nerve traction injury.

We present a unique case of combined MCN and radial nerve injury following open subpectoral tenodesis, supported by multimodal imaging—including ultrasound evidence of a rope-effect tendon-nerve interaction—and confirmed surgically.

## Case report

A 64-year-old right-handed male automotive mechanic and recreational karate practitioner presented with chronic anterior shoulder pain that interfered with overhead mechanical tasks and martial arts training. Magnetic resonance imaging demonstrated degenerative intra-articular pathology of the LHB consistent with superior labral anterior to posterior lesions. Mild medial subluxation of the LHB within the bicipital groove was also noted.

Three weeks after an ultrasound-guided corticosteroid injection (1 cc of triamcinolone acetonide, Trigon), the patient experienced an acute “pop” and sudden deformity. A confirmatory ultrasound demonstrated a complete LHB rupture with marked distal retraction.

The patient was concerned about regarding loss of strength and performance during sports activity. Given his high functional demands as a mechanic and karate athlete, surgical treatment was performed as soon as the diagnosis was established, which was two weeks after the tendon rupture. The patient underwent open subpectoral biceps tenodesis using a suture anchor (BioComposite SwiveLock 9 x 19,5 mm, Arthrex). Intraoperatively, the tendon stump was found deeply retracted into the muscular portion of the arm and displayed significant degeneration. Extensive scar tissue was present, which made identification of the tendon difficult. Hohmann retractors were positioned laterally along the humeral shaft, whereas Farabeuf retractors were used medially within the surgical field to facilitate soft-tissue retraction and visualization of the tenodesis site. Standard fixation was completed without apparent technical complication.

Immediately after surgery, the patient exhibited weak elbow flexion, complete paralysis of wrist and finger extension, weak supination, and sensory loss over the lateral forearm and radial nerve distribution, a pattern consistent with combined MCN and radial nerve palsy, raising concern for brachial plexus involvement.

Magnetic resonance neurography demonstrated diffuse T2 hyperintensity along the MCN and radial nerve branches without evidence of nerve discontinuity, accompanied by surrounding soft-tissue inflammatory changes, findings compatible with neuritic injury but insufficient to localize the mechanical cause (Fig. 1). High-resolution ultrasound provided the decisive diagnostic information, revealing the MCN coursing deep to the tenodesed tendon, which sharply angulated and compressed the nerve in a characteristic rope-effect configuration; the nerve appeared flattened at the entrapment point with proximal and distal fusiform swelling and loss of fascicular definition, and dynamic assessment showed worsening compression during humeral external rotation (Fig. 2). To our knowledge, this represents the first published case in which ultrasound clearly demonstrates both MCN entrapment and a rope-effect mechanism in conjunction with simultaneous radial nerve injury.

**Figure 1:**
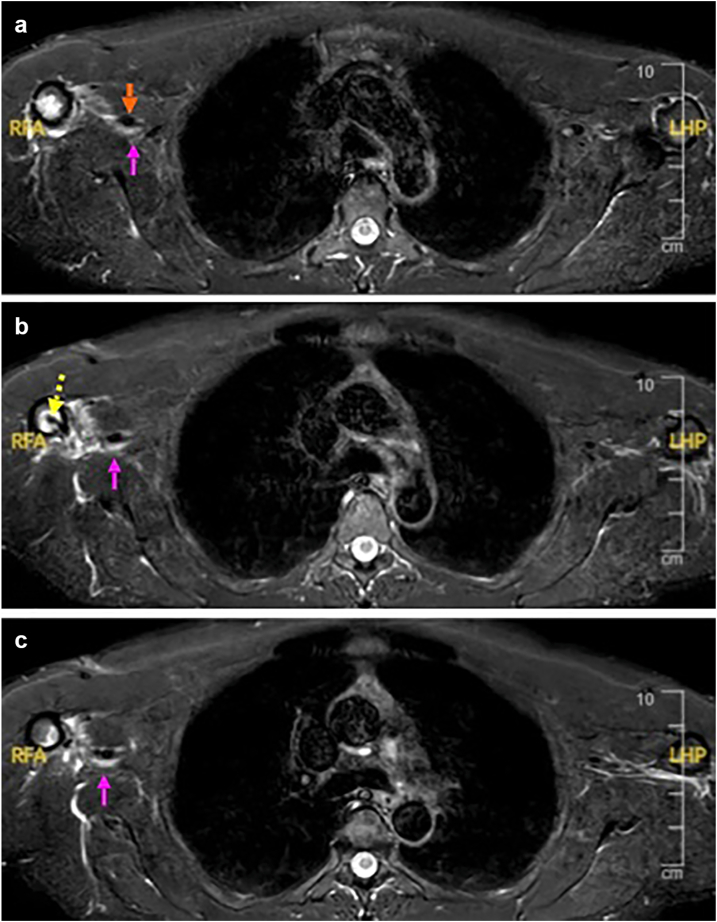
MRI of brachial plexus. Axial fat-suppressed fluid sensitive consecutive images obtained in a caudal plane to clavicle and lateral border of pectoralis minor muscle (key anatomic landmark of brachial plexus that serves at the landmark for the branches). (**a-c**) Show linear increased signal intensity (*pinkarrow*) posterior to right axillary artery (*orangearrow*) corresponding to main terminal branches of brachial plexus and consistent with nerve injury (this finding is not observed on contralateral side). The main terminal branches have a typical relationship with the axillary artery with the musculocutaneous nerve and radial nerve located in posterior quadrant respect axillary artery, like our case. Tenodesed long head of the biceps tendon at medial humeral quadrant yellow arrow in figure (**b**) and inflammatory changes in surgical bed. *MRI*, magnetic resonance imaging.

**Figure 2:**
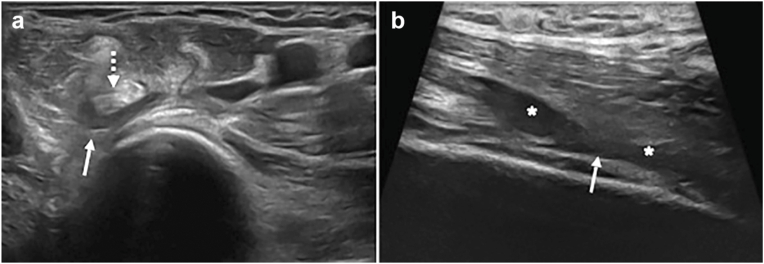
US images obtained in a proximal plain of the right arm abducted and externally rotated. (**a**) Transverse US view shows the tenodesed tendon (*dashedarrow*) and the musculocutaneous nerve (*whitearrow*) located deep to the tendon and with flattened, compressed and sharply angulated morphology. (**b**) Longitudinal US view show flattened nerve at the entrapment tendon point (*arrow*) and proximal and distal nerve hypoechoic swelling with loss of fascicular definition. *US*, ultrasonography

A conventional electromyography of the right upper limb muscles was performed six weeks after surgery, including the deltoid, biceps, triceps, palmaris muscle, brachioradialis, extensor digitorum communis, extensor pollicis, and first dorsal interosseous. The study showed marked acute denervation of the biceps, with no motor unit potentials recordable in that muscle. There was also acute denervation in muscles innervated by the radial nerve throughout its course, although motor unit potentials were still present, albeit poorly configured, especially in the distal radial-innervated muscles. Muscles innervated by the axillary nerve showed chronic neurogenic changes with only limited signs of reinnervation. Muscles innervated by the ulnar nerve were normal. Electromyography supported a dual injury pattern, showing severe MCN axonotmesis with absent biceps motor units, acute denervation of brachioradialis and wrist and finger extensors, absent radial sensory conduction, and preservation of median and ulnar nerve function, confirming the coexistence of focal mechanical MCN compression and traction-related radial neuropathy. Given the severity and persistence of the deficit, and considering the patient's occupational and athletic demands, early revision surgery was undertaken. Intraoperative findings confirmed the imaging diagnosis: the tenodesed LHBs tendon had been inadvertently medialized, crossing directly over the MCN, which was found entrapped and compressed beneath the tendon with a clear indentation at the contact site, reproducing the rope-effect mechanism; compression increased with humeral external rotation. The interference screw was removed, the tenodesis reversed, and extensive external neurolysis of the MCN was performed (Fig. 3).

**Figure 3:**
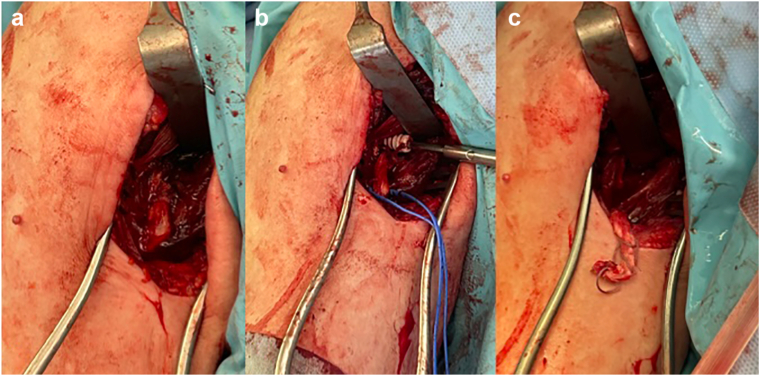
Intraoperative images of the surgical revision. (**a**) Appearance of the tenodesed long head of the biceps tendon entrapping the musculocutaneous nerve beneath it. (**b**) Removal of the interference screw. (**c**) Appearance of the musculocutaneous nerve, constricted but without disruption, after removal of the interference screw.

Follow-up electromyography at 3 months demonstrated progressive reinnervation with return of motor unit potentials in the biceps and brachioradialis, reduction of fibrillation potentials, and improving radial motor conduction, although radial sensory responses remained absent. The patient has fully recovered brachial biceps function as well as wrist extension and finger extension. A neurophysiological study of the right upper limb was performed 10 months after the initial surgery. The findings were suggestive of significant improvement. The ulnar nerve was normal, and motor conduction through the MCN was normal with only mild hypoexcitability. There was persistent radial nerve involvement but with only scarce signs of acute/active denervation. Signs of reinnervation were observed in the distal radial-innervated muscles, with motor unit potentials now present in these muscles, whereas in the previous study, they had been poorly configured. Overall, radial nerve involvement was moderate.

At the current follow-up, almost 1 year after the initial surgery, the patient has resumed an almost normal daily life. He subjectively estimates his functional recovery at approximately 90%. Based on the neurophysiological findings, further neurological improvement may still be expected over time.

## Discussion

This case demonstrates a unique dual-mechanism nerve injury after biceps tenodesis. Most MCN injuries reported are transient and attributed to traction or retraction.^6^^,^^9^ Only 1 prior publication has documented true entrapment of the nerve beneath a tenodesed tendon.^4^ Our case represents the first documented instance combining MCN entrapment with radial nerve traction injury.

Anatomical and cadaveric studies show that the MCN crosses near the tenodesis zone and that its distance from the humerus increases significantly with external rotation,^2^^,^^3^^,^^8^ supporting the rationale that medialized fixation or improper arm positioning may predispose to nerve injury. Radial nerve involvement likely resulted from traction transmitted through chronically retracted tendon fibers, a mechanism consistent with prior descriptions of traction-induced brachial plexopathy.^1^^,^^5^^,^^7^

The significance of early multimodal imaging is supported by literature noting that ultrasound and magnetic resonance neurography are highly informative for localizing nerve entrapment and distinguishing mechanical from neuritic lesions.^3^^,^^8^

## Pearls and pitfalls

This case highlights that tenodesis after corticosteroid-injection–related tendon rupture should be considered hazardous, particularly when surgery is not performed acutely. The complication described in this article may have been partially related to the distorted anatomy and scar tissue encountered in this delayed autotenotomy setting. In this case, approximately two weeks had elapsed between the diagnosis of the LHBs rupture and the surgical procedure. During surgery, a substantial amount of scar tissue was already present, which significantly increased the technical difficulty of the intervention and made anatomical dissection more demanding. Based on our experience with this case, we would discourage this procedure when it is not performed in the acute setting, particularly in cases of corticosteroid-injection–related tendon rupture, where tendon quality and local tissue conditions may be compromised.

In view of the complication observed in this case, we strongly discourage the use of pointed-tip retractors in this setting. Moreover, even prolonged retraction with blunt retractors should be avoided or minimized, particularly when surgical exposure is difficult or the anatomy is distorted by scar tissue.

The therapeutic benefit in these cases may be limited, while the potential risks are considerable. Therefore, the decision to proceed with surgical treatment can be controversial and should be carefully weighed on an individual basis, taking into account the patient's functional demands, timing from rupture, tendon quality, and the potential for neurovascular complications.^8^

## Disclaimers:

Funding: No funding was disclosed by the authors.

Conflicts of interest: The authors, their immediate family, and any research foundation with which they are affiliated have not received any financial payments or other benefits from any commercial entity related to the subject of this article.

Patient consent: Obtained.
